# Muscarinic receptor M3 activation promotes fibrocytes contraction

**DOI:** 10.3389/fphar.2022.939780

**Published:** 2022-09-06

**Authors:** Pauline Henrot, Edmée Eyraud, Elise Maurat, Sophie Point, Guillaume Cardouat, Jean-François Quignard, Pauline Esteves, Thomas Trian, Pierre-Olivier Girodet, Roger Marthan, Maéva Zysman, Patrick Berger, Isabelle Dupin

**Affiliations:** ^1^ Univ-Bordeaux, Centre de Recherche Cardio-thoracique de Bordeaux, INSERM U1045, Pessac, France; ^2^ CHU de Bordeaux, Hôpital du Haut-Lévêque, Services des Maladies Respiratoires, et Explorations Fonctionnelles Respiratoires, Pessac, France; ^3^ Inserm, Centre de Recherche Cardio-thoracique de Bordeaux, U1045 and CIC1401, Pessac, France

**Keywords:** fibrocyte, COPD, M3, cholinergic, contraction

## Abstract

Fibrocytes are monocyte-derived cells able to differentiate into myofibroblasts-like cells. We have previously shown that they are increased in the bronchi of Chronic Obstructive Pulmonary Disease (COPD) patients and associated to worse lung function. COPD is characterized by irreversible airflow obstruction, partly due to an increased cholinergic environment. Our goal was to investigate muscarinic signalling in COPD fibrocytes. Fibrocytes were isolated from 16 patients with COPD’s blood and presence of muscarinic M3 receptor was assessed at the transcriptional and protein levels. Calcium signalling and collagen gels contraction experiments were performed in presence of carbachol (cholinergic agonist) ± tiotropium bromide (antimuscarinic). Expression of M3 receptor was confirmed by Western blot and flow cytometry in differentiated fibrocytes. Immunocytochemistry showed the presence of cytoplasmic and membrane-associated pools of M3. Stimulation with carbachol elicited an intracellular calcium response in 35.7% of fibrocytes. This response was significantly blunted by the presence of tiotropium bromide: 14.6% of responding cells (*p* < 0.0001). Carbachol induced a significant contraction of fibrocytes embedded in collagen gels (13.6 ± 0.3% versus 2.5 ± 4.1%; *p* < 0.0001), which was prevented by prior tiotropium bromide addition (4.1 ± 2.7% of gel contraction; *p* < 0.0001). Finally, M3-expressing fibrocytes were also identified *in situ* in the peri-bronchial area of COPD patients’ lungs, and there was a tendency to an increased density compared to healthy patient’s lungs. In conclusion, around 1/3 of COPD patients’ fibrocytes express a functional muscarinic M3 receptor. Cholinergic-induced fibrocyte contraction might participate in airway diameter reduction and subsequent increase of airflow resistance in patients with COPD. The inhibition of these processes could participate to the beneficial effects of muscarinic antagonists for COPD treatment.

## Introduction

Chronic obstructive pulmonary disease (COPD) is a severe and prevalent disease, currently the third leading cause of death in the world, linked to inhaled particles exposure such as tobacco smoke (Lozano et al., 2012). It encompasses both bronchial obstruction and alveolar destruction, however its pathophysiology is complex and remains not fully elucidated (Agustí and Hogg, 2019). Airway obstruction in particular, involves increased airway contraction associated to bronchial remodelling with peri-bronchiolar fibrosis. Increased cholinergic tone is also an important feature of this disease, as well as the only reversible component (Brusasco, 2006). Acetylcholine, mainly secreted by the vagal nerve, promotes airway contraction through muscarinic receptors activation on bronchial smooth muscle cells, as well as mucus secretion from epithelial cells and submucosal glands (Barnes, 1993). Among all five muscarinic receptors, the Gq-coupled M3 receptor is the primary subtype responsible for airway contraction (Gosens et al., 2006). Tiotropium bromide, a long-acting bronchodilator inhibiting M1, M2 and M3, is the reference treatment in the therapeutic class of long-acting muscarinic antagonists (LAMAs) (Tashkin et al., 2008; D’Urzo et al., 2018).

Fibrocytes are a distinct population of monocyte-derived cells, classically described as co-expressing haematopoietic and progenitor cell markers, therefore bearing features of both macrophages and fibroblasts (Bucala et al., 1994). They are able to migrate to injured organs and to differentiate into myofibroblasts-like cells. In the field of respiratory diseases, they have initially been described as biomarkers for idiopathic pulmonary fibrosis (Moeller et al., 2009). Their implication has then been strongly suggested in other lung diseases such as asthma (Schmidt et al., 2003; Wang et al., 2021), cystic fibrosis (Kasam et al., 2020), and more recently Chronic Obstructive Pulmonary Disease (COPD) (Dupin et al., 2018). We have notably found that fibrocytes were increased in the blood of COPD patients during exacerbations (Dupin et al., 2016), as well as in COPD bronchial tissues, associated with worse lung function (Dupin et al., 2019). Altogether, fibrocytes seem to play a significant role in bronchial remodelling.

Our goal was to investigate the role of muscarinic signalling in COPD fibrocytes, by assessing the presence and functionality of the M3 receptor.

## Materials and methods

### Cell culture

After written informed consent, blood samples were obtained from patients with COPD within the Cohort of Bronchial obstruction and Asthma (COBRA, Ethics committee number: 2008-A00294-51/1), sponsored by the French National Institute of Health and Medical Research, INSERM). Fibrocytes were isolated from peripheral blood samples of patients with COPD (n = 16) and cultured for 14 days to reach full differentiation, as previously described (Dupin et al., 2016). Briefly, non-adherent non-T (NANT) cells were purified from peripheral blood mononuclear cells (PBMCs) separated from the whole blood and incubated for 1 week in Dulbecco’s Modified Eagle’s Medium (DMEM) (Fisher Scientific, Illkirch-Graffenstaden, France) supplemented with 20% Fetal Calf Serum (FCS) (Biowest, Nuaillé, France), followed by 1 week in serum-free medium. Fibrocyte characteristic spindle-shaped morphology was checked by using phase-contrast microscopy (Nikon, Amsterdam, Pays-Bas). For all experiments, cells were used at full differentiation (*i.e*. after 12–14 days in culture). Bronchial smooth muscle cells (SMCs) were obtained from subjects with normal FEV1 and FEV1/FVC, as previously described (Dupin et al., 2019; Celle et al., 2022).

### Real-time Ca^2+^ fluorescence confocal imaging

Differentiated fibrocytes were bathed in physiological saline solution (in mM: 130 NaCl, 5.6 KCl, two CaCl_2_, one MgCl_2_, 11 glucose, eight HEPES, pH = 7.4) and incubated with the fluo-4/AM probe (1.5 µM, Biotium/Clinisciences, Nanterre, France) during 30 min at 37°C. After 15 min washout, fluorescence was observed under a Nikon d-Eclipse C1 confocal scanning microscope. If applicable, tiotropium bromide (Sigma/Merck, Lyon, France) was added in the medium 2 h before the measurements, and thapsigargin (1 µM in DMSO, Sigma) 15 min before measurement. For the condition without extracellular calcium, no calcium was added in the bathing solution and the calcium chelator EGTA at 0.2 mM was also added. Images were acquired every 10 s during 3 min and baseline fluorescence was considered mean of the first three acquisitions. Carbachol (carbamoyl chloride, Sigma, from 10^−4^ to 10^−6^ M) was added into the wells of the plate between the third and the fourth acquisition. Adenosine triphosphate (ATP, Sigma) 10^−4^ M was used as a positive control. The amplitude of the calcium rise was determined as the maximum Δ(F/F0) reached during the recording time. A calcium response was considered positive when Δ(F/F0) was ≥0.1 (Cussac et al., 2020). For each patient and each condition, 50 individual cells were analysed. Experiments were performed in fibrocytes cultured from seven different patients with COPD, and for each experiment and condition, two wells were analysed.

### Contraction assay in collagen gel

Contraction assay was performed as previously described (Matsumoto et al., 2007). Briefly, a cell suspension of 10^6^ fibrocytes per mL in DMEM 0.1% BSA was mixed (1:1 ratio) with an ice-cold collagen suspension (for one gel: Collagen I rat tail, Enzo Life Sciences, Villeurbanne, France, 3 mg/ml; NaOH, 8.33 mM/L; Phosphate Buffered Saline (PBS), qsp 300 µL). The resulting suspension was immediately cast into a 24-wells plate and allowed to polymerize during 45 min at 37°C. Each gel contained 300,000 fibrocytes for a final collagen concentration of 1.5 mg/ml. After polymerization, gels were submerged with 0.4 ml of DMEM 0.1% BSA and kept at 37°C overnight. The day after, inhibitors (if applicable) were directly added in the well prior to the experiment (blebbistatin 10 µM (Sigma) or Y-27632 10 µM (Sigma), 15 min prior to the experiment; tiotropium bromide 10^−6^ M (Sigma), 2 h prior to the experiment). The gel was then carefully detached from the culture well and transferred into a 6-wells plate containing 3 ml of Krebs-Hepes solution (in mM: NaCl 118.4; KCl 4.7; MgSO_4_ 1.2; NaHCO_3_ four; KH_2_PO_4_ 1.2; Hepes 10; Glucose 6). Gels were stimulated no more than 5 minutes after detachment, with KCl 80 mM, carbachol 10^−4^ M or vehicle (PBS). Images of the gels were acquired every 30 s during 20 min with a fixed camera (Huawei) and analysed with the software ImageJ (version 1.53). Experiments were performed in fibrocytes cultured from five different patients with COPD, and for each experiment and condition, two gels were analysed. For analysis, the whole gel surface was measured before stimulation and after 1, 5, 10 and 20 min. Percentage of contraction is expressed as 100—(resulting gel surface divided by initial gel surface).

### Immunohistochemistry

Immunostaining of lung samples was performed as previously described (Dupin et al., 2019). Briefly, samples were embedded in paraffin, and 2.5 µm-thick sections were stained with a rabbit anti-FSP1 polyclonal antibody (Agilent) and a mouse anti-CD45 monoclonal antibody (BD Biosciences) with appropriate fluorescent secondary antibodies, and a mouse monoclonal Alexa 647-coupled anti-M3 antibody (Santa Cruz). Sections were imaged using a Nanozoomer 2.0HT slide scanner (Hamamatsu Photonics). Identification and quantification of FSP1^+^ CD45^+^ M3^+^ cells (triple-positive cells) was performed through automated image analysis using the software Fiji (NIH). The density of FSP1^+^ CD45^+^ M3^+^ cells was defined by the ratio between the numbers of triple-positive cells in the lamina propria divided by the lamina propria area. Tissue area and cell analysis were all performed in a blinded fashion.

### Statistical analyses

Values are presented as mean with standard deviation or median (95% confidence interval). Statistical significance, defined as *p* < 0.05, was analysed by Fisher’s exact test for the comparison of proportions, by the two-sided independent *t*-test or 2-way Anova for variables with a parametric distribution, and by the Wilcoxon test and Mann–Whitney test for variables with a nonparametric distribution.

## Results

### Differentiated fibrocytes from patients with COPD express the muscarinic M3 receptor

Clinical characteristics of patients with COPD are shown in Table 1.

**TABLE 1:** Clinical characteristics of patients with COPD.

*n*	16
Age (years)	66.8 ± 6.8
Female/Male (n)	6/10
Current/Former smokers (n)	7/9
Smoking history (pack years)	44.1 ± 15.2
GOLD stages: I/II/III/IV (n)	0/11/5/0
GOLD stages: A/B/C/D (n)	3/7/0/6
FEV1 (% pred)	54.4 ± 9.7
FEV1/FVC (%)	55.7 ± 6.7
TLC (% pred)	106.7 ± 11.1
RV (% pred)	138.3 ± 24.7
TLCO (% pred)	56.4 ± 11.0
6-min walking test: distance (m)	497 ± 76

Data are mean ± SD (or otherwise specified). Abbreviations: FEV1: Forced Expiratory Volume in 1 s; FVC: forced vital capacity; TLC: total lung capacity; RV: residual volume; TLCO: transfer lung capacity of carbon monoxide; pred: predicted.

**FIGURE 1:**
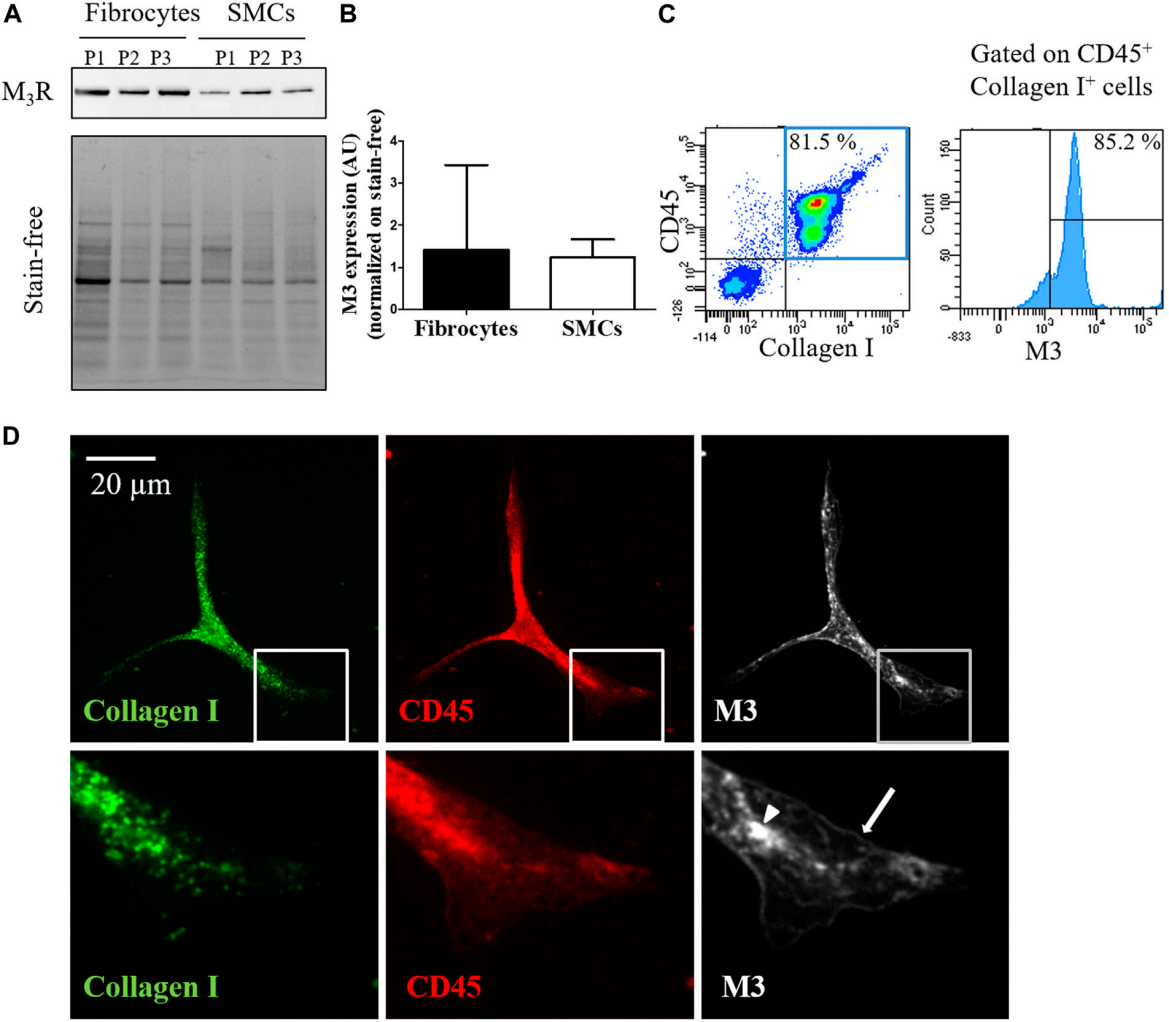
Fibrocytes from patients with COPD express the muscarinic M3 receptor. **(A)**. Presence of M3 receptor (M_3_R) is assessed in cultured fibrocytes from patients with COPD or in bronchial smooth muscle cells (SMCs) by Western blot. Total protein load is shown with the stain-free image. **(B)**. quantification of M_3_R expression normalized on stain-free. No significant difference is found between fibrocytes and SMCs (*p* = 0.40). **(C)**. M3 receptor is identified by flow cytometry in fibrocytes isolated from the blood of COPD patients. In the represented experiment, 85.2% of fibrocytes (double-positive CD45-Collagen I cells, blue square) express M3. **(D)**. Representative confocal photographs show the expression of M3 receptor (white) evidenced in the cytoplasm (arrow head) and at the surface (arrow) of a fibrocyte (CD45^+^ Collagen I^+^ fusiform cell).

By real-time quantitative Polymerase Chain Reaction, we identified the expression of transcripts of all five muscarinic receptors in cultured fibrocytes extracted from the blood of COPD patients, including M3 (Supplementary Figure 1A,B). Expression of M3 at the protein level was confirmed by Western blotting in cultured fibrocytes, using bronchial SMCs as a positive control (Figure 1A). M3 expression was comparable between SMCs and fibrocytes (Figure 1B). Moreover, using flow cytometry, 59.7% (±15) of fibrocytes (identified through a double CD45-Collagen I staining) were found to express the M3 receptor after 7 days in culture (Figure 1C). Immunocytochemistry showed the presence of cytoplasmic and membrane-associated pools of M3 in cultured fibrocytes (Figure 1D), showing that some M3 proteins were localized at the cell surface.

### Stimulation of M3 with the muscarinic agonist carbachol elicits a calcium response in fibrocytes

We then assessed the intracellular calcium response to a muscarinic stimulation in differentiated fibrocytes from patients with COPD. We used ATP 10^−4^ M as a positive control, as well as performed the same experiment in bronchial SMCs from control subjects in order to compare the magnitude of the calcium response. We stimulated cultured fibrocytes with different concentrations of the cholinergic agonist carbachol, ranging from 10^−4^ M to 10^−6^ M, and found that the treatment with 10^−4^ M carbachol elicited the highest percentage of responding cells, in a dose-response manner (Supplementary Figure S2A). This concentration was thus chosen for the subsequent experiments.

**FIGURE 2:**
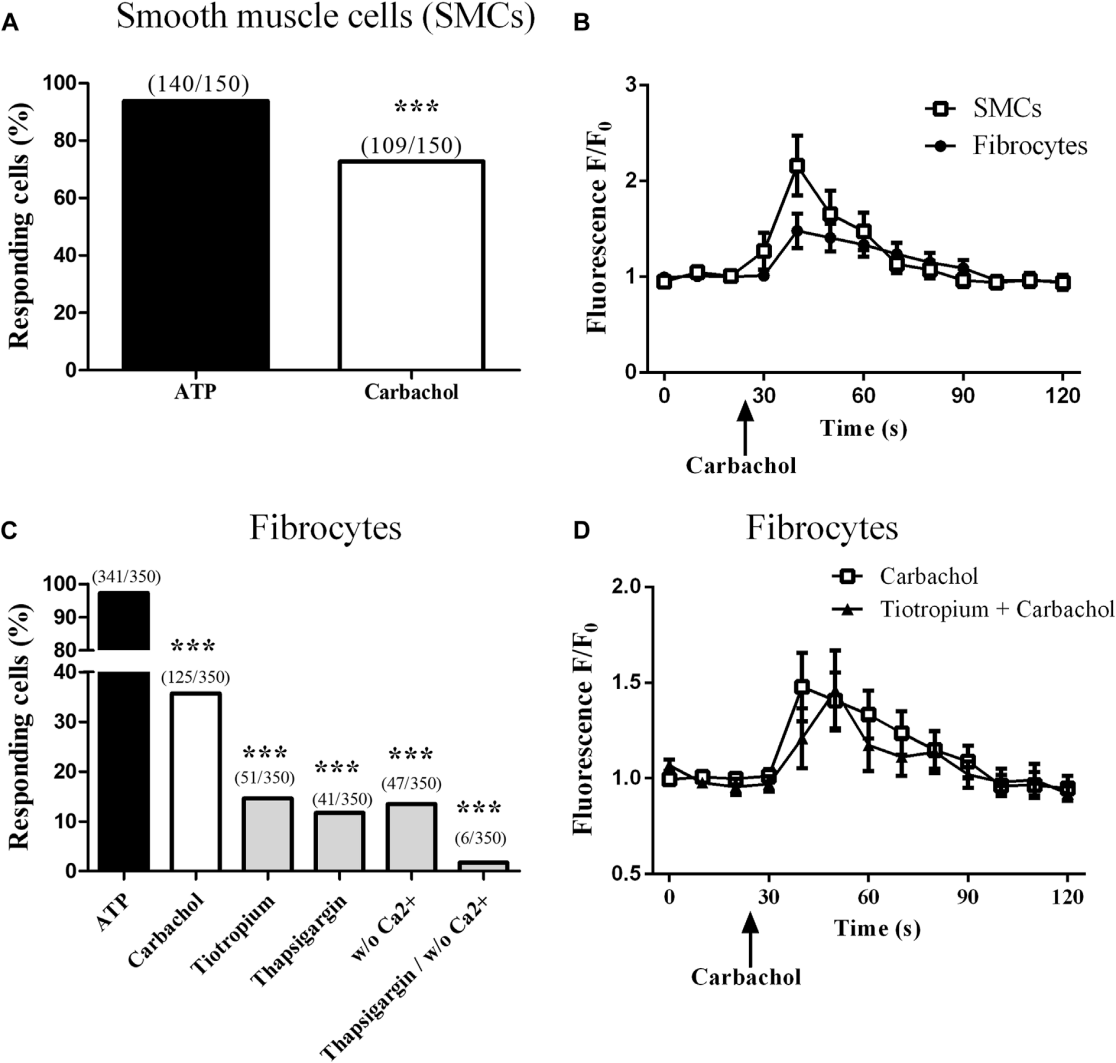
Muscarinic M3 receptor expressed on cultured fibrocytes from patients with COPD is functional. Variations of relative cytosolic Ca^2+^ concentration were monitored by fluorescence video microscopy in fluo4-loaded fibrocytes and smooth muscle cells (SMCs). **(A)**. Proportion of responding cells to 10^−4^ M carbachol for control SMCs. **(B)**. Variations of relative cytosolic Ca^2+^ concentration against time (mean ± SD) in responding cells, for SMCs (squares) and fibrocytes (circles). **(C)**. Proportion of responding cells for COPD fibrocytes stimulated with 10^−4^ M ATP and with 10^−4^ M carbachol, in the absence or the presence of tiotropium bromide 10^−6^ M, thapsigargin 10 µM and with or without extracellular Ca^2+^. **(D)**. Variations of relative cytosolic Ca^2+^ concentration against time (mean ± SD) in responding cells, for fibrocytes stimulated with carbachol with (triangles) or without (squares) prior addition of tiotropium bromide 10^−6^ M. Significant difference ***p < 0.0001, otherwise no significant difference.

Stimulation of bronchial SMCs with 10^−4^ M carbachol elicited an intracellular calcium response in 59.8% (±1.4) of the cells (Figure 2A). Mean peak amplitude of responding cells was 2.25 (±0.19) (Supplementary Figure S2D). In contrast, stimulation of fibrocytes with carbachol 10^−4^ M elicited an intracellular calcium response in 37.5% of fibrocytes (Figure 2C), showing that muscarinic receptors were functional. Mean peak amplitude of responding cells was 1.64 (±0.02) (Supplementary Figure S2B). Typical mean curves of responding cells for fibrocytes and SMCs are shown as an illustration in Figure 2B.

### Carbachol-induced calcium response is significantly blunted by muscarinic antagonist

We then sought to assess the downstream implicated pathways. Prior depletion of endoplasmic reticulum (ER) calcium stores by 1 µM thapsigargin significantly inhibited the calcium response (percentage of responding cells: 11.7%; *p* < 0.0001, Figure 2C), consistent with calcium release from the ER, downstream of the Gq-coupled receptor M3. Depletion of extracellular calcium also inhibited the calcium response (percentage of responding cells: 13.4%; *p* < 0.0001, Figure 2C), suggesting an additional store-operated calcium entry mechanism (Hogan and Rao, 2015). Consistent with the previous findings, combining both conditions almost completely inhibited the calcium response (percentage of responding cells: 1.7%; *p* < 0.0001, Figure 2C).

Finally, prior addition of the muscarinic M2/M3 antagonist tiotropium bromide (10^−6^ M) significantly blunted the calcium response to carbachol: 14.6% of responding cells (*p* < 0.0001, Figure 2C). As tiotropium was added 2 h before the measurement and is known to dissociate very rapidly from M2 (Barnes et al., 1995), this suggests that the calcium response was muscarinic M3-specific. The 10^−6^ M concentration was chosen as the concentration eliciting the highest inhibition of calcium response to carbachol 10^−4^ M in a dose-response experiment where tiotropium doses ranged from 10^−9^ to 10^−6^ M (data not shown) and consistent with literature (Arai et al., 2010; Kruse et al., 2012). Typical mean curve of responding cells to carbachol and tiotropium 10^−6^ M is shown in Figure 2D. Mean peak amplitude of responding cells was not significantly different between all groups (Supplementary Figure S2B).

Of note, tiotropium 10^−6^ M also significantly inhibited the calcium response to carbachol in SMCs, with 24.2% of responding cells (*p* < 0.0001, Supplementary Figure S2C,D).

### Carbachol stimulation promotes fibrocyte contraction

Then, we investigated the consequences of M3 activation on COPD fibrocytes. Long-term stimulation (5 days) of cultured fibrocytes with carbachol did not influence cell differentiation, as assessed by the percentage of *α*-smooth muscle actin (α-SMA)^+^ cells (Supplementary Figure S3A) and vimentin-positive cells (Supplementary Figure S3B). Short-term stimulation (overnight) did not influence fibrocyte migration in transwell assay (Supplementary Figure S3C). Given that muscarinic pathways promote contraction in smooth muscle cells, we sought whether carbachol influenced fibrocytes contraction.

**FIGURE 3:**
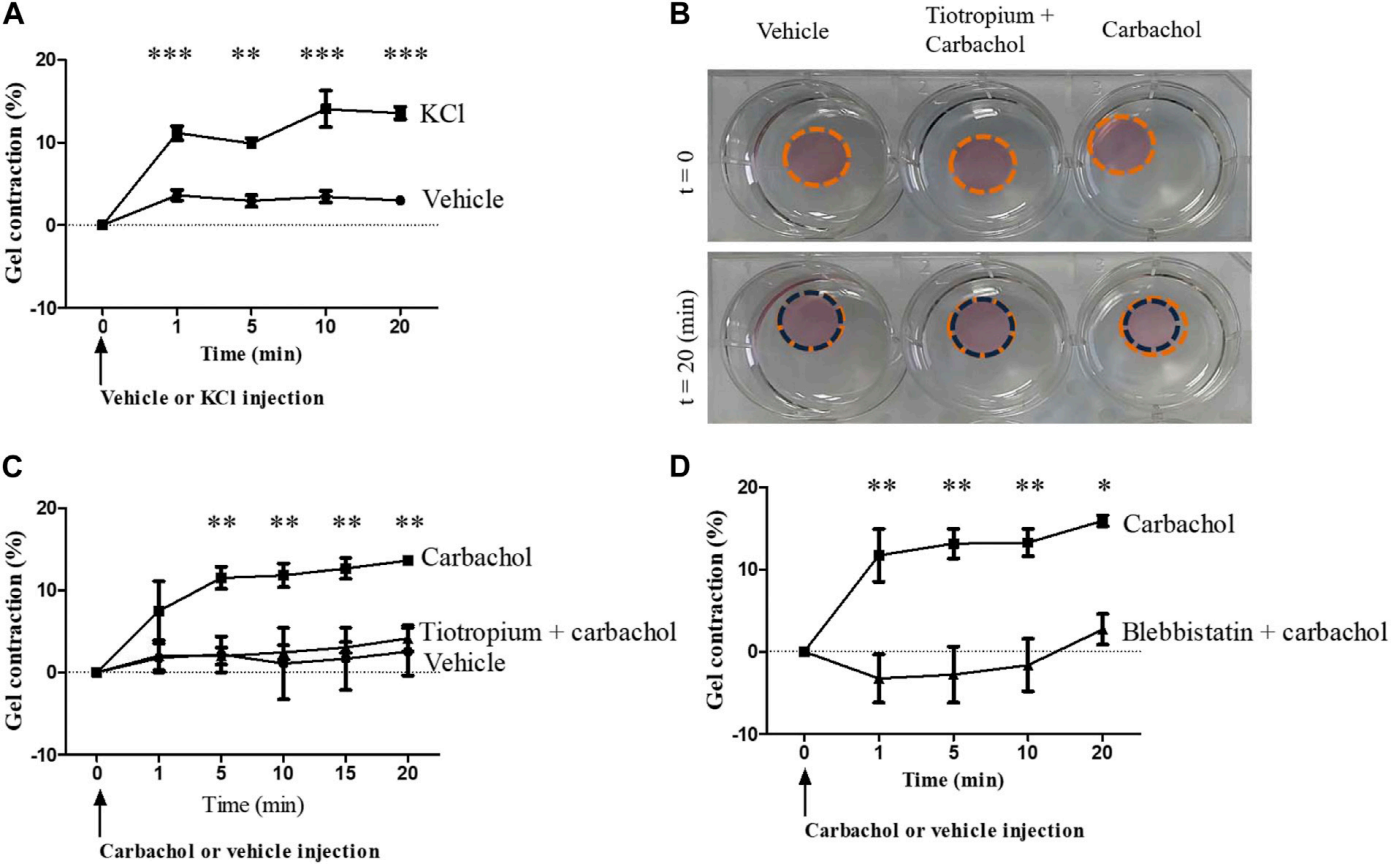
Muscarinic M3 receptor activation promotes COPD fibrocytes contraction. **(A)**. Significant contraction of fibrocytes-embedded collagen gels stimulated with KCl 80 mM (squares) compared to control vehicle (circles). Percentage of gel contraction is presented against time. **(B)**. Aspect of fibrocytes-embedded collagen gels stimulated with control vehicle or with carbachol 10^−4^ M, with or without prior tiotropium bromide 10^−6^ M addition, before and 20 min after the beginning of the experiment. **(C)**. Significant contraction of fibrocytes-embedded collagen gels stimulated with carbachol 10^−4^ M (squares) compared to control vehicle (circles) or tiotropium bromide 10^−6^ M + carbachol 10^−4^ M (triangles). **(D)**. Significant inhibition of carbachol-induced gel contraction (squares) when incubating collagen gels with blebbistatin 10 µM prior to carbachol stimulation (triangles). Significant difference *p < 0.05, **p < 0.01, ***p < 0.001, otherwise no significant difference.

Fibrocytes were embedded in collagen gels and their contractile potential was first investigated using KCl 80 mM, leading to a maximum contraction of 14.0 ± 3% of the gel compared to vehicle (*p* < 0.001, Figure 3A). Then, we stimulated fibrocytes with carbachol and observed a significant contraction compared to control: 13.6 ± 0.3% of maximal gel contraction versus 2.5 ± 4.1%; *p* < 0.0001 (Figures 3B,C). As a mean of comparison, stimulation of bronchial SMCs embedded in collagen gels with carbachol led to a maximal gel contraction of 22.5 ± 10% (Supplementary Figure S3D). As a negative control, we also stimulated collagen gels without cells with carbachol and observed no contraction (data not shown).

### Carbachol-induced gel contraction is significantly inhibited by muscarinic antagonist

Prior addition of tiotropium bromide significantly inhibited the carbachol-induced contraction of the fibrocyte-embedded gel, resulting in a contraction of 4.1 ± 2.7%; *p* < 0.0001 (Figures 3B,C), showing that this contraction was M3-dependant. Moreover, the use of the myosin II inhibitor blebbistatin (10 µM) led to a significant inhibition of carbachol-induced gel contraction (*p* < 0.01, Figure 3D), suggesting that downstream activated pathway depends on actomyosin contractility (Kovács et al., 2004). Of note, the use of the Rho-kinase inhibitor Y-27632 (10 µM) also led to a significant inhibition of carbachol-induced gel contraction (data not shown).

### M3-expressing fibrocytes are present in COPD lungs *in situ*

**FIGURE 4:**
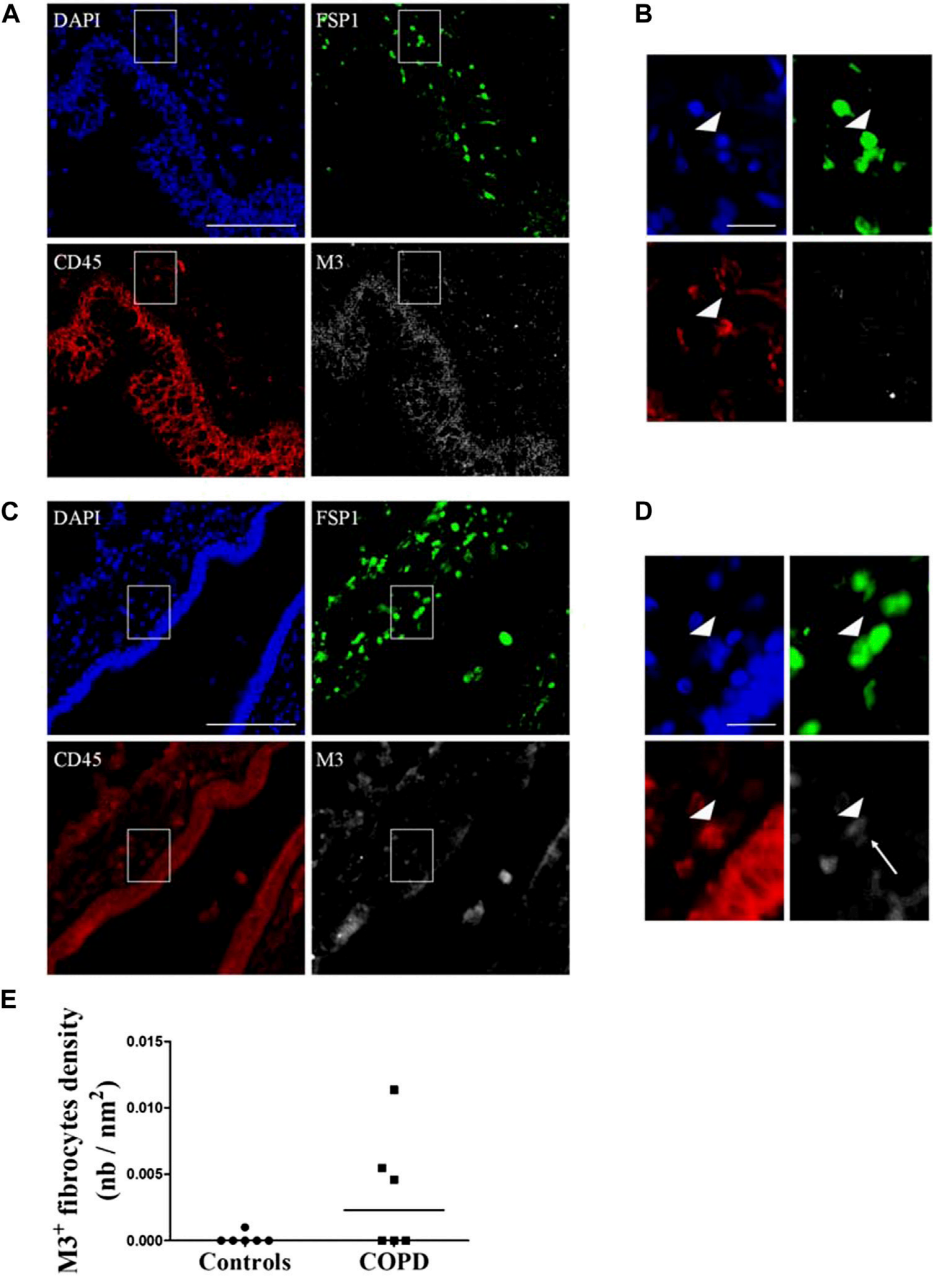
Identification of M3-expressing fibrocytes in the lungs of COPD patients and healthy subjects. Peri-bronchial fibrocytes are identified as double-positive FSP1+ (green) CD45^+^ (red) cells. M3 staining is represented in white and nuclei are stained with DAPI (blue). **(A)** representative triple immunostaining of a healthy subject’s lung tissue. Scale bar, 100 µm. **(B)**. Close-up view of the sub-epithelial region indicated in A (white square). Arrowhead indicate a fibrocyte, identified as a double positive FSP1-CD45 cell, without M3 expression. Scale bar, 20 µm. **(C)** representative triple immunostaining of a COPD patient’s lung tissue. Scale bar, 100 µm. **(D)**. Close-up view of the sub-epithelial region indicated in C (white square). Arrowhead indicate a fibrocyte, identified as a double positive FSP1-CD45 cell, expressing M3 (white arrow). Scale bar, 20 µm. **(E)**. Quantification of M3^+^ fibrocytes densities as represented by the number of peri-bronchial triple-positive cells divided by the area of lamina propria analyzed.

We sought whether M3-expressing fibrocytes could be identified in airways of COPD patients and whether their density was different from that of healthy controls airways. We performed a triple immunostaining for CD45, FSP1 and M3 in lungs from healthy controls and COPD patients in residual lung samples from a previously published study (“Fibrochir” study (Dupin et al., 2019)). Clinical characteristics for healthy subjects and COPD patients are shown in Supplementary Table S2. Overall, most M3-expressing fibrocytes were identified in the peri-bronchial area of COPD patients’s lungs samples and only few could be observed in healthy subjects’ lungs. Representative images of healthy subjects’ and COPD patients’ lungs are shown in Figure 4A-D. Quantification of the peri-bronchial densities of M3-expressing fibrocytes showed a tendency to an increased density in COPD patient’s samples compared to healthy subjects, although the difference was not statistically significant (*p* = 0,18, Figure 4E).

**FIGURE 5:**
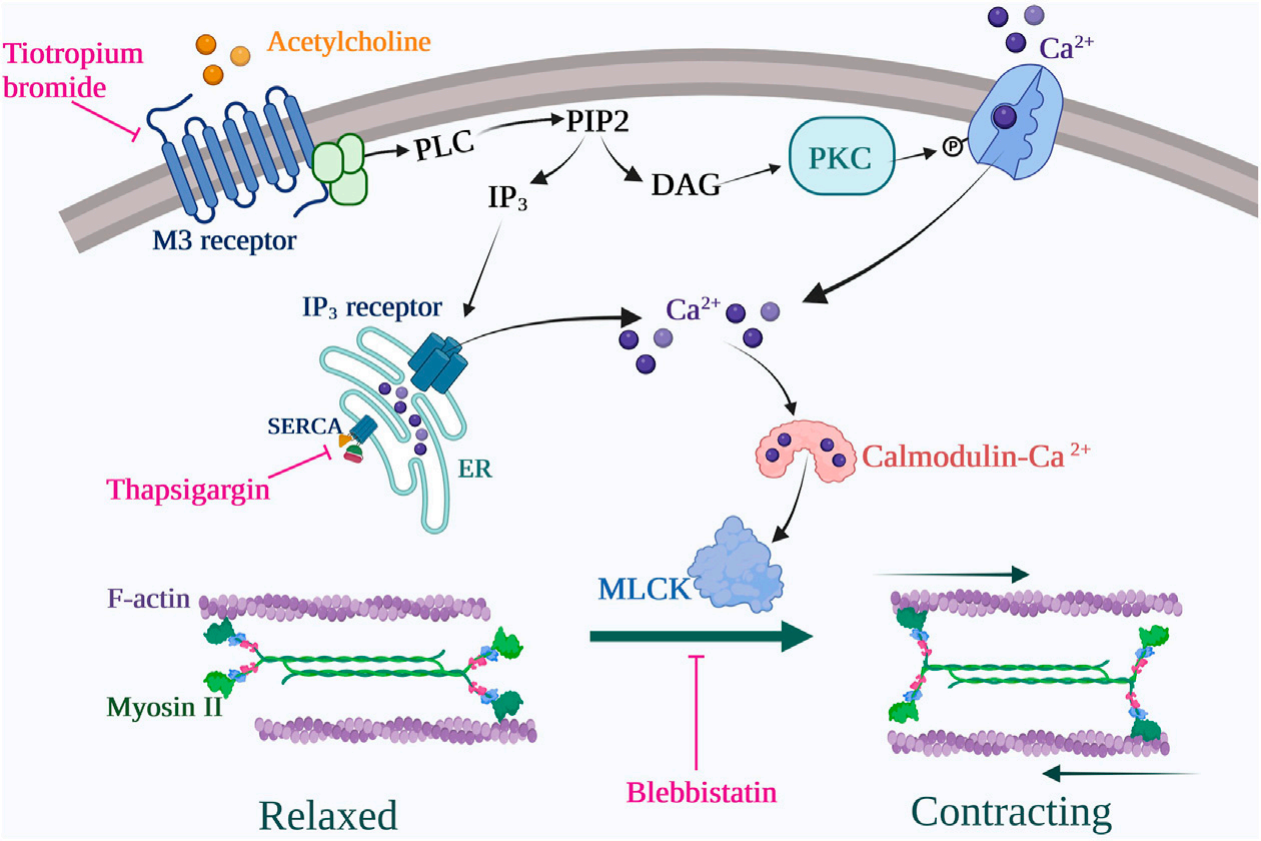
Proposed signalling pathway for COPD fibrocytes stimulated with cholinergic/muscarinic agonists. Muscarinic M3 receptor activation leads to intracellular Ca^2+^ increase via emptying of endoplasmic reticulum (ER) stores as well as secondary extracellular Ca^2+^ uptake (store-operator calcium entry). Cytosolic Ca^2+^ rise leads to acto-myosin dimerization via myosin light chain kinase (MLCK) activation, thus leading to cell contraction. These processes are inhibited at the receptor level by tiotropium bromide (M2/M3 antagonist) use, at the endoplasmic reticulum level by thapsigargin use and at the contraction level by blebbistatin use. Created with BioRender.com.

Together, our data indicate that M3-expressing fibrocytes can be identified *in situ* mostly in COPD lungs. Our results also show that stimulation of differentiated COPD fibrocytes with carbachol induces cellular contraction via the Gq-coupled M3 receptor activation, leading to intracellular calcium release and actomyosin contraction. We propose a model for the signalling pathways and cellular processes activated by muscarinic agonists in COPD fibrocytes in Figure 5.

## Discussion

Collectively, our data indicate that around one third of fibrocytes from COPD patients’ blood express a functional muscarinic M3 receptor and that the activation of this receptor leads to cellular contraction. These findings introduce fibrocytes as potential additional cellular players in COPD airway contraction.

Increased cholinergic tone is classically targeted by short- and long-acting muscarinic antagonists (SAMA and LAMA), such as the M1/M2/M3 antagonist tiotropium bromide, considered as a predominant M1/M3 antagonist as it dissociates very rapidly from M2. All LAMAs (aclidinium, glycopyrronium, tiotropium, and umeclidinium) have been shown equivalent in terms of clinical efficacy (Ismaila et al., 2015). However, interestingly, it is not the case *in vitro*: in a recent study on bronchial rings, tiotropium and umeclidinium effects remained stable for at least 9 h whereas aclidinium and glycopyrronium displayed less stable inhibitory effects, with a progressive loss of inhibition at submaximal concentrations (Naline et al., 2018). Therefore, it would be interesting in a future study to compare their effects on carbachol-mediated fibrocytes contraction. Besides its inhibitory effects on airway contraction and beneficial effects on dyspnoea and lung function, tiotropium is also associated with general beneficial effects such as reduction in number of exacerbations and subsequent hospitalizations, and effectiveness of pulmonary rehabilitation (Karner et al., 2012). These clinical data point towards a role for the cholinergic system in the inflammatory or pro-fibrotic processes of bronchial remodelling, in addition to airway contraction, although this has never been formally demonstrated to date. In this context, fibrocytes appear as an interesting cellular target to investigate, being implicated in both processes. Indeed, fibrocytes participate to ECM remodelling and regulation, directly or through paracrine signalling (Kleaveland et al., 2014). They also take part in inflammatory processes, as they are able to secrete pro-inflammatory cytokines (Dupin et al., 2018) as well as interact with immune cells, particularly CD8^+^ T lymphocytes, in the lungs (Afroj et al., 2021). Finally, we have demonstrated here for the first time that fibrocytes were able to contract upon cholinergic stimulation. Altogether, the deleterious action of fibrocytes could be related to either phenomenon.

Our study has some limitations. First, we did not have access to fibrocytes from healthy subjects’ blood, which prevents us from drawing any definite conclusion concerning the specificity of M3 activation in fibrocytes from COPD patients compared to healthy subjects. Moreover, our main findings were obtained *in vitro* and can not necessarily be translated *in vivo*. However, we have observed M3-expressing fibrocytes *in situ*, and their density seemed increased, although not in a statistically significant manner (possibly due to the small sample size), in lungs from COPD patients compared to healthy controls. Future studies could focus on deciphering the role of M3-mediated fibrocyte contraction in COPD pathophysiology, for example in 3D co-culture of SMCs and fibrocytes such as in (Lin et al., 2014).

In our study, amplitude of fibrocytes-embedded collagen gel contraction was small. Besides the fact the M3 receptor is expressed by only a portion of cultured fibrocytes, this small contraction is probably also due to the fact that fibrocytes are not professional contractile cells, unlike SMCs. Of note, only one other study investigated the contractile power of fibrocytes embedded in collagen gels an found a basal cellular contraction of 20% over 4 days (Ling et al., 2019). We do believe however it is contraction and not collagen remodelling, as blebbistatine, an inhibitor of myosin-II-specific ATPase, completely inhibited carbachol-induced gel contraction; moreover, the rapidity of carbachol effect (within a few minutes) also advocates for contraction, as collagen remodelling would be slower.

Altogether, given the slight, although significant, contraction observed in our study, it is unlikely that fibrocytes are major contributors of COPD airway contraction. Nevertheless, two local consequences could arise from the subclinical fibrocyte-driven increase in airway tone. First, it might contribute to increase SMCs contraction, through an increased environmental rigidity, which was evidenced in a recent study (Polio et al., 2019). Second, increased rigidity might further enhance ECM deposition by resident myofibroblasts by mechano-transduction pathways (Tschumperlin et al., 2018), thus contributing to enhance peribronchial fibrosis.

To conclude, targeting lung fibrocytes in COPD might improve respiratory function and other important outcomes. Pre-clinical studies targeting fibrocyte recruitment or their differentiation into the lungs, via therapeutic strategies directed against chemokines and their receptors (Berger et al., 2019), could help us to determine the full implication of fibrocytes in COPD pathophysiology.

## Data Availability

The original contributions presented in the study are included in the article/Supplementary Material, further inquiries can be directed to the corresponding author.
