# Supplementary material for: Muscarinic receptor M3 activation promotes fibrocytes contraction
Source: Front Pharmacol. 2022 Sep 6;13:939780. doi: 10.3389/fphar.2022.939780 (PMC9485632; doi:10.3389/fphar.2022.939780)
Supplement: Supplementary file 1 [file DataSheet1.docx]

Supplementary Material

# Supplementary methods

*Cell culture – bronchial smooth muscle cells*

TUBE was a collection of human bronchial tissue obtained from thoracic lobectomy surgery for nodule or cancer (pN0) and was sponsored by the University hospital of Bordeaux, which includes its own local ethic committee (CHUBX 2020/54). According to the French law and the MR004 regulation, patients received an information form, allowing them to refuse the use of their surgical samples for research. SMCs were isolated from specimens and cultured as previously described [1].

*RNA extraction and real-time quantitative PCR (qPCR)*

Total RNAs were extracted from fibrocytes (n=6) or SMCs (n=2) using RNeasy kit, following the manufacturer’s recommendations (Qiagen). RNA (1 μg) was reverse-transcribed using the iScriptTM cDNA synthesis kit (Bio-Rad). cDNA samples were then analyzed by qPCR using iQTM SYBR® Green supermix through the MyiQTM real-time PCR detection system (Bio-Rad). M1 to M5 primers were purchased from Sigma-Aldrich and are listed in Table S1.

*Western blot*

Total cells lysis for fibrocytes (n=3) or SMCs (n=3) was performed using a RIPA lysis buffer (Sigma-Aldrich). Total cellular extracts were loaded onto a 10 % SDS-PAGE gel (Bio-Rad, Hercules, CA), transferred onto nitrocellulose membrane and incubated with a primary antibody against M3 receptor (ab126168, Abcam - RRID:AB_11129778). HRP-coupled secondary antibody was used for revelation using ChemiDoc imaging instrument (Bio-Rad). Protein expressions were normalized using total loading protein intensity (Stain-free system Bio-Rad).

*Flow cytometry*

Fibrocytes (n=7) were detached using Accutase (Fisher Scientific) incubated at room temperature, fixed and permeabilized with the kit Cytofix/Cytoperm (eBiosciences) and incubated with fluorescence-coupled primary antibodies against CD45 (BD Biosciences) or Collagen I (Abcam), or primary antibody against M3 receptor (ab126168, Abcam) and specific fluorescent secondary antibody (Santa Cruz). Data were acquired using a Canto II 4-Blue 2-Violet 2-Red laser configuration (BD Biosciences).

*Immunocytochemistry*

Adherent fibrocytes were fixed with cold 4% paraformaldehyde, washed three times with phosphate buffered saline (PBS), permeabilized with Triton 0.1% in PBS, saturated with 4 % Bovine Serum Albumin (BSA) in PBS and stained with primary antibodies directed against CD45 (clone HI-30, Sony Biotechnology), Collagen I (Abcam) and the M3 receptor (ab126168, Abcam). Secondary antibodies attached to fluorescent probes were used and images were acquired using an inverted confocal scanning microscope (Nikon D-Eclipse C1, Nikon).

**Reference**

1. Celle, A.; Esteves, P.; Cardouat, G.; Beaufils, F.; Eyraud, E.; Dupin, I.; Maurat, E.; Lacomme, S.; Ousova, O.; Begueret, H.; et al. Rhinovirus Infection of Bronchial Epithelium Induces Specific Bronchial Smooth Muscle Cell Migration of Severe Asthmatic Patients. *J Allergy Clin Immunol* **2022**, S0091-6749(22)00148-8, doi:10.1016/j.jaci.2022.01.022.

# Supplementary Figures and Tables

**Supplementary Table 1: primers for muscarinic receptors**

|  | **Forward** | **Reverse** |
| --- | --- | --- |
| **M1** | CAATCACTGGCTGTGCCTCTC | TGAAGAGGGGTCTGTAGGGTC |
| **M2** | CCCTGGGCCATTCCAAAGAT | TCTTCACAATCTTGCGGGCT |
| **M3** | TGCTACATCAACAGCACCGT | TGCGCGCTTGTGAAAAATGA |
| **M4** | GCTCACTCGGGTTTCCATGA | GAAGGGAACCTGGCTGACTC |
| **M5** | ACATATCGGGCCAAGCGTACTC | AACTGGATCTGGCACTCATCCA |

**Supplementary Table 2: clinical characteristics of healthy subjects and COPD patients for lung samples**

Data are mean ± SD (or otherwise specified). Abbreviations: FEV1: Forced Expiratory Volume in 1 sec; FVC: Forced Vital Capacity; TLC: Total Lung capacity; RV: Residual Volume; TLCO: Transfer Lung capacity of Carbon monoxide; pred: predicted. *, significant difference.

|  | Healthy subjects | COPD patients | p-value |
| --- | --- | --- | --- |
| n | 6 | 6 |  |
| Age (years) | 59.2 ± 6.2 | 73.5 ± 5.4 | 0.002* |
| Female (%) | 33.3 % | 50 % | 1.00 |
| Non smokers/ Former smokers / Current smokers (n) | 1/3/2 | 0/6/0 | 0.14 |
| Smoking history (pack years) | 28.3 ± 18.6 | 52 ± 22.8 | 0.09 |
| FEV1 (% pred) | 93.3 ± 19.5 | 70 ± 9.7 | 0.04* |
| FEV1/FVC (%) | 0.82 ± 0.2 | 0.64 ± 0.1 | 0.005* |
| TLC (% pred) | 107.3 ± 13.8 | 91.2 ± 9.8 | 0.39 |
| RV (% pred) | 115.7 ± 29.1 | 125.2 ± 24.9 | 0.59 |
| TLCO (% pred) | 76.2 ± 27.2 | 66.2 ± 16.1 | 0.78 |

**Supplementary Figure 1: differentiated fibrocytes express all five muscarinic receptors.**

**
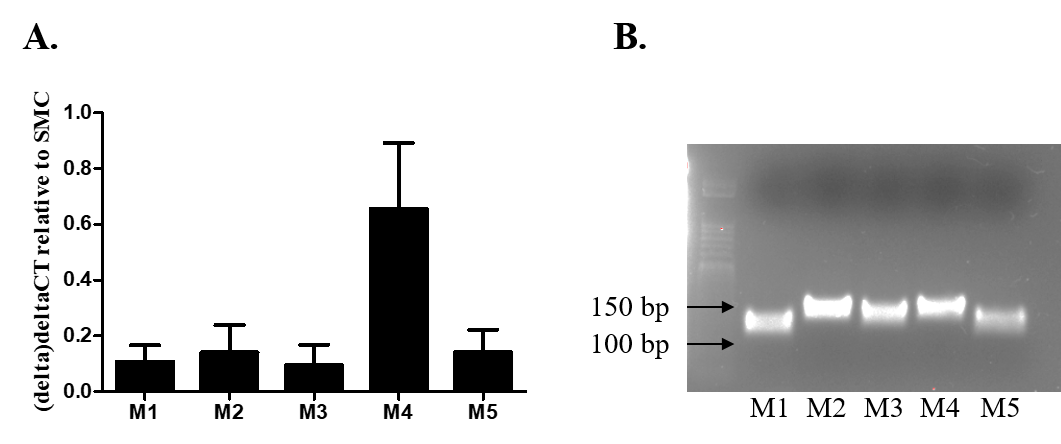
**

A: Presence of muscarinic transcripts (M1 to M5) is assessed in cultured fibrocytes from COPD patients or in bronchial smooth muscle cells (SMCs) from control subjects by qPCR. Results are shown relative to SMCs transcripts expression.

B: Bands for qPCR products for M1 to M5 transcripts are visualized on 1% agarose gel.

**Supplementary Figure 2: calcium signalling in cultured fibrocytes and smooth muscle cells (SMCs).**

**
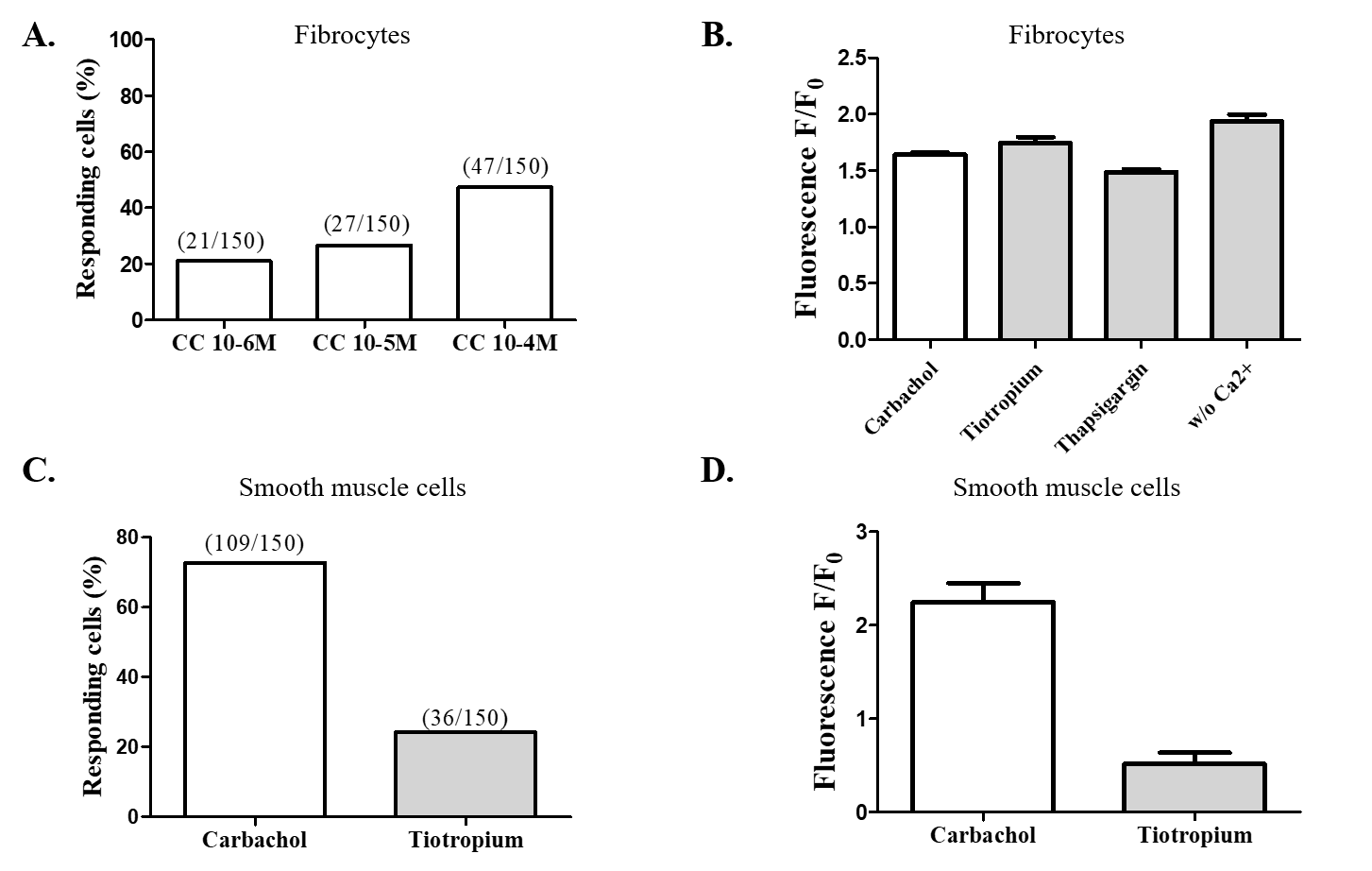
**

Variations of relative cytosolic Ca2+ concentration were monitored by fluorescence video microscopy in fluo4-loaded fibrocytes and SMCs.

A : Mean ± SEM percentage of responding cells for fibrocytes stimulated with increasing doses of carbachol (CC), ranging from 10^-6^ to 10^-4^M.

B: Mean ± SEM peak amplitude of responding cells, stimulated with 10^-4^M carbachol, without or with prior addition of tiotropium bromide 10^-6^M or thapisgargin 10 µm, or without extra-cellular Ca^2+^ addition.

C: Mean ± SEM percentage of responding cells to 10^-4^M carbachol for control SMCs, without or with prior tiotropium bromide 10^-6^M addition.

B: Mean ± SEM peak amplitude of responding cells for SMCs stimulated with 10^-4^M carbachol without or with prior addition of tiotropium bromide 10^-6^M.

**Supplementary Figure 3: effect of carbachol stimulation on cultured fibrocytes and smooth muscle cells from COPD patients.**

**
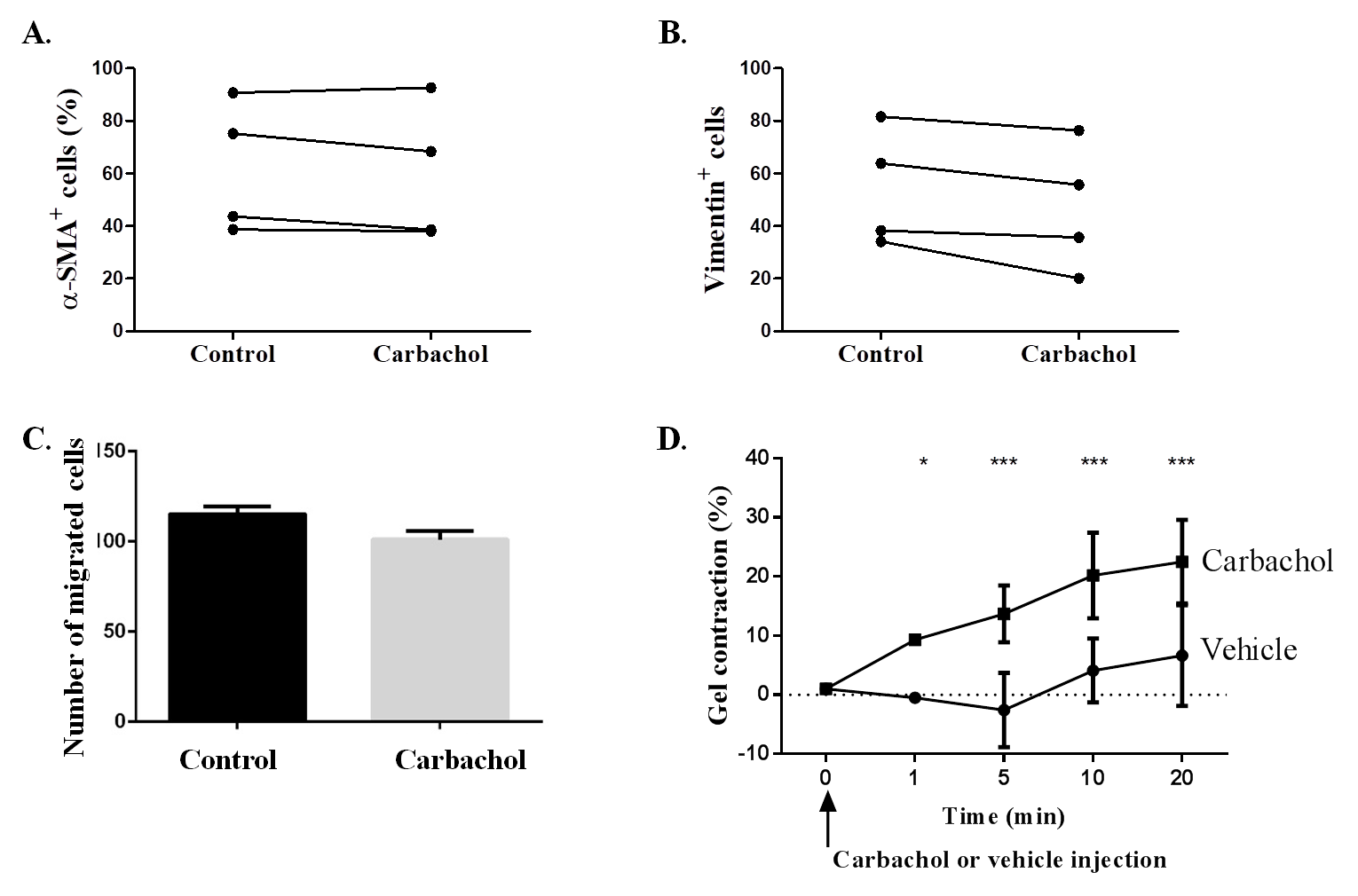
**

A, B: Fibrocyte differentiation is assessed by the percentage of α-smooth muscle actin in the absence (control) or in the presence of 10^-4^M carbachol for 5 days. No significant difference was seen in either case.

C: Fibrocyte migration is assessed in transwell in the absence (black bar) or in the presence of 10^-4^M carbachol (grey bar) for 12 hours. No significant difference was observed.

D: Significant contraction of smooth muscle cells-embedded collagen gels stimulated with carbachol 10^-4^M (squares) compared to control vehicle (circles). Significant differences *p < 0.05, ***p < 0.001, ****p < 0.0001 using 2-way Anova.

**
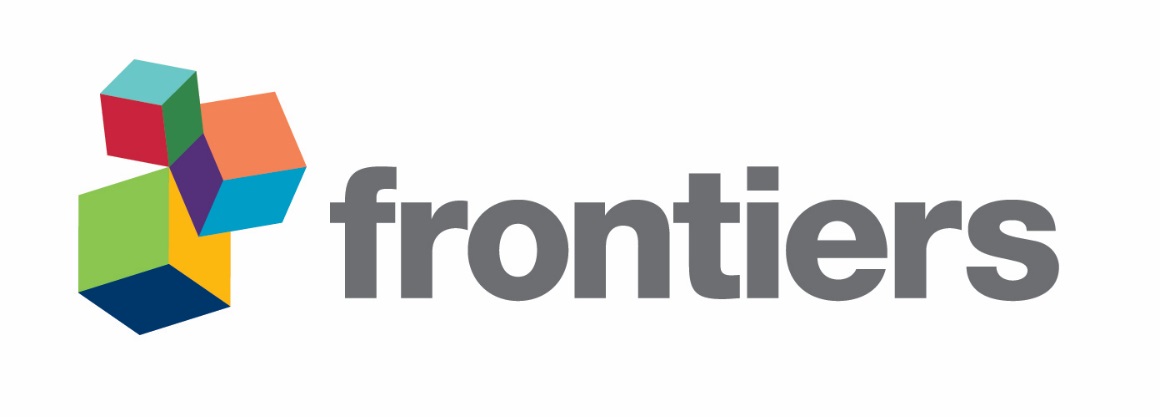
**
